# Increased genetic diversity of ADME genes in African Americans compared with their putative ancestral source populations and implications for Pharmacogenomics

**DOI:** 10.1186/1471-2156-15-52

**Published:** 2014-05-01

**Authors:** Jing Li, Xingzhen Lao, Chao Zhang, Lei Tian, Dongsheng Lu, Shuhua Xu

**Affiliations:** 1 Max Planck Independent Research Group on Population Genomics, Chinese Academy of Sciences and Max Planck Society (CAS-MPG) Partner Institute for Computational Biology, Shanghai Institutes for Biological Sciences, Chinese Academy of Sciences, Shanghai 200031, China; 2 School of Life Science and Technology, China Pharmaceutical University, Nanjing 210009, China

**Keywords:** ADME genes, African Americans, Drug response heterogeneity, Genetic diversity, Genetic admixture

## Abstract

**Background:**

African Americans have been treated as a representative population for African ancestry for many purposes, including pharmacogenomic studies. However, the contribution of European ancestry is expected to result in considerable differences in the genetic architecture of African American individuals compared with an African genome. In particular, the genetic admixture influences the genomic diversity of drug metabolism-related genes, and may cause high heterogeneity of drug responses in admixed populations such as African Americans.

**Results:**

The genomic ancestry information of African-American (ASW) samples was obtained from data of the 1000 Genomes Project, and local ancestral components were also extracted for 32 core genes and 252 extended genes, which are associated with drug absorption, distribution, metabolism, and excretion (ADME) genes. As expected, the global genetic diversity pattern in ASW was determined by the contributions of its putative ancestral source populations, and the whole profiles of ADME genes in ASW are much closer to those in YRI than in CEU. However, we observed much higher diversity in some functionally important ADME genes in ASW than either CEU or YRI, which could be a result of either genetic drift or natural selection, and we identified some signatures of the latter. We analyzed the clinically relevant polymorphic alleles and haplotypes, and found that 28 functional mutations (including 3 missense, 3 splice, and 22 regulator sites) exhibited significantly higher differentiation between the three populations.

**Conclusions:**

Analysis of the genetic diversity of ADME genes showed differentiation between admixed population and its ancestral source populations. In particular, the different genetic diversity between ASW and YRI indicated that the ethnic differences in pharmacogenomic studies are broadly existed despite that African ancestry is dominant in Africans Americans. This study should advance our understanding of the genetic basis of the drug response heterogeneity between populations, especially in the case of population admixture, and have significant implications for evaluating potential inter-population heterogeneity in drug treatment effects.

## Background

Many factors such as age, enzyme induction or inhibition, and diseases can affect enzyme activity. Variations in the DNA sequence of enzyme-encoding genes can abolish, reduce, or increase the activity of an enzyme. The genetic variations in the genes involved in the absorption, distribution, metabolism, and excretion (ADME) of drugs are therefore essential factors for the efficacy and safety of drugs in the human body [1,2]. Generally, ADME enzymes are composed of Phase I metabolizing enzymes (such as the cytochrome P450 enzymes), Phase II metabolizing enzymes (such as arylamine N-acetyltransferase), and drug transporters (including the ATP binding cassette proteins) [3]. Previous studies highlighted the contributions of both environmental and, in particular, genetic factors to variations in the activity of ADME proteins [4,5]. Some functional polymorphisms have therefore been reported in ADME genes that allow the classification of individuals into intermediate, rapid, and slow metabolized groups, and the broad distribution of drug responses might increase the risk of drug therapy when the therapeutic window is narrow [6]. The careful assessment of the contributions of ADME genetic variations to the efficacy and safety of drugs is an important task for the development of clinical pharmacogenetic studies.

Population studies have revealed that ethnic differences occur in the frequency of genetic variants [7,8], and that significant genetic differences in the ADME genes between different populations could lead to therapeutic failure, or adverse drug responses. For example, the intronic SNP located at *CYP3A5*, known as “CYP3A5*3”, results in a nonfunctional protein, and occurs at a frequency of ~40% among African Americans, ~90% among Caucasians, and ~65% among Asians [9]. Additional important ADME genes, such as *CYP2C9*, *CYP2C19*, *CYP2D6*, and *NAT2*, also have significantly different frequencies of genetic variants that may lead to different drug dose requirements of different ethnic groups [10,11]. For example warfarin, an anticoagulant, has the highest dose requirements in African-Americans, the lowest dose requirements in Asians, and intermediate requirements in Caucasian populations [12]. Since the populations in developing countries rely mainly on the US FDA or European Medical Agency guidelines for dosing instructions, a comprehensive understanding of the inter-ethnic differences in the ADME genes is therefore critical to guide more effective global drug prescriptions [13].

African Americans are well known admixed from Africans and Europeans [14]. As the largest minority group in the United States, African Americans have received significant attention in pharmacogenetic studies. However, little is known about the influence of admixture on genetic heterozygosity and haplotype diversity, and how it may directly implicate the heterogeneity of drug responses. Furthermore, limited pharmacogenetic data are available on African populations [15], and so systematic comparisons of the patterns and magnitudes of diversity of ADME genes between African and African-American populations would benefit the drug responses of Africans, and facilitate future inter-ethnic investigations of drug metabolism.

We compared the genetic diversity of ADME genes (including 32 core genes and 252 extended genes) and that of non-ADME genes which were randomly selected from the list of known genes in the genome in African-American, African, and European populations. We then investigated the genetic architectures of ADME genes and searched for the factors that could influence the genetic diversity of ADME genes in the three populations. Further, we identified functional mutations with highly differential allele frequencies and compared the distributions of haplotypes clinically defined in each ADME core gene among the three populations. Finally, we explored the mechanism of higher genetic diversity of ADME genes in admixed population like the African American compared with that in its ancestral source populations.

## Results

### Ancestral origins of ADME genes in African Americans

**Figure 1:**
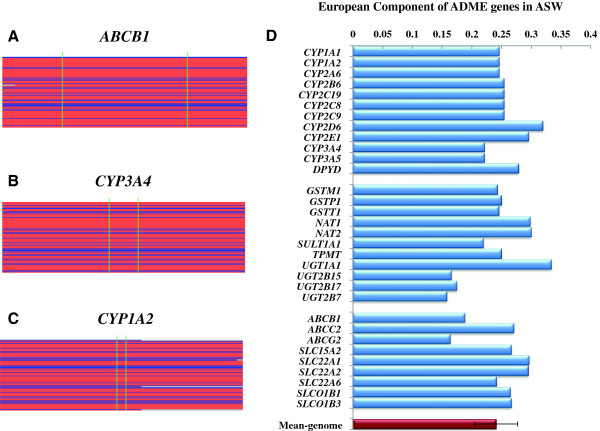
**Ancestral origins of ADME genes in African Americans.** The examples of ancestral origins of three ADME core genes in African Americans: **(A)***ABCB1*, **(B)***CYP3A4*, **(C)***CYP1A2*, where each box has 122 rows representing the diploid sequences of 61 individuals. Blue colored fragments mean originating from European, red means originating from Africa, and gray means unknown component. The start and end positions of genes are plotted at corresponding locations using green bars, and the up- and down-stream 100 kb regions are also included in the figures. **(D)** The percentage of local European genetic components in ASW for 32 ADME genes. The red bar at the bottom of panel represents the average percentage of European genetic components from the whole genome, while the error bars represent the SD of the percentages. Note that for *CYP2E1*, *GSTM1*, *SULT1A1*, *UGT1A1*, *UGT2B15*, *ABCG2*, *SLC22A1*, and *SLCO1B1* the percentages are average values, while for the remaining genes they are consistent values (see Additional file 2: Figure S2).

Estimating the local admixture proportions of genes could help not only to understand the genetic differences between admixed and ancestral source populations, but also to investigate the natural selection that has occurred since admixture [16]. Here, we directly extracted the local ancestry information of each ADME gene from the dataset provided by the 1000 Genomes Project. In Additional file 1: Figure S1 and Additional file 2: Figure S2, data showed that different ADME genes in ASW exhibited highly variable origins, even for the same individual at different regions. For example, an ASW individual (NA19625), who is presented as first two rows in each box, exhibited two haplotypes of the *ABCB1* gene that originated from Europeans (Figure 1A), two haplotypes of the *CYP3A4* gene that originated from Africans (Figure 1B), and haplotypes of the *CYP1A2* gene that originated from Europeans and Africans separately (Figure 1C). Detailed descriptions of the local ancestral information of all 61 African American subjects at the ADME 32 core genes are shown in Additional file 3: Table S1. It is likely that individuals may exhibit significant differences in drug metabolism due to the heterogeneity of ancestral origins. Because of this, the analysis of the ancestral origins of ADME genes in admixed populations (such as ASW) is important to understand the high heterogeneity of drug responses in these populations.

We further examined the local ancestry contributions from Europeans to African Americans in 32 ADME core genes and 252 ADME extended genes (Figure 1D and Additional file 4: Table S2). The average contribution of European ancestry is 24.1% (SD = 0.036), based on autosomal data, which is consistent with previous studies [14,17]. However, the European genetic contributions varied from 15.8% (*UGT2B7*) to 33.4% (*UGT1A1*) in 32 ADME core genes, and from 15.3% (*SULT1C2*) to 34.6% (*ABCG1*) in the 252 ADME extended genes. In summary, none of the European ancestries of these 284 ADME genes significantly deviated from the average value of whole autosomes (<3 SD, 13.3%, and approximately 34.9% European ancestral component). These results did not support strong natural selection of the ADME genes in African-Americans since admixture [16].

### The diversity patterns of ADME genes in African Americans

In pharmacogenetic studies, allele frequency, heterozygosity, and haplotype diversity have been commonly used as indicators of heterogeneity of the drug response. To further investigate the influence of admixture on ADME genes in African Americans, we examined the fluctuations in heterozygosity and haplotype diversity of each 10 kb bin spanning the 32 ADME core genes (Additional file 2: Figure S2). It is clear that heterozygosity and haplotype diversity have similar trends in the three populations. In most examples, the diversity pattern of ASW is closer to YRI, and both are significantly higher than CEU. Furthermore, heterozygosity and haplotype diversity vary much more than the local ancestral components, where the genetic diversity could significantly change in neighboring 10 kb bins, while similar ancestral fragments could span hundred thousand base pairs. It is likely that the genetic diversity patterns of an admixed population were affected not only by local ancestral proportions, but also by the patterns of its ancestral source populations.

To further investigate the influence of ancestral contributions on the genetic diversity of admixed populations, we carried out correlation analysis between the observed and expected allele frequencies of ASW, which was calculated using the following formula:

$$f^{\mathrm{ASW},\exp}=f^{\mathrm{CEU}}.\rho^{\mathrm{Eur}}+f^{\mathrm{YRI}}.\left(1-\rho^{\mathrm{Eur}}\right)$$

**Figure 2:**
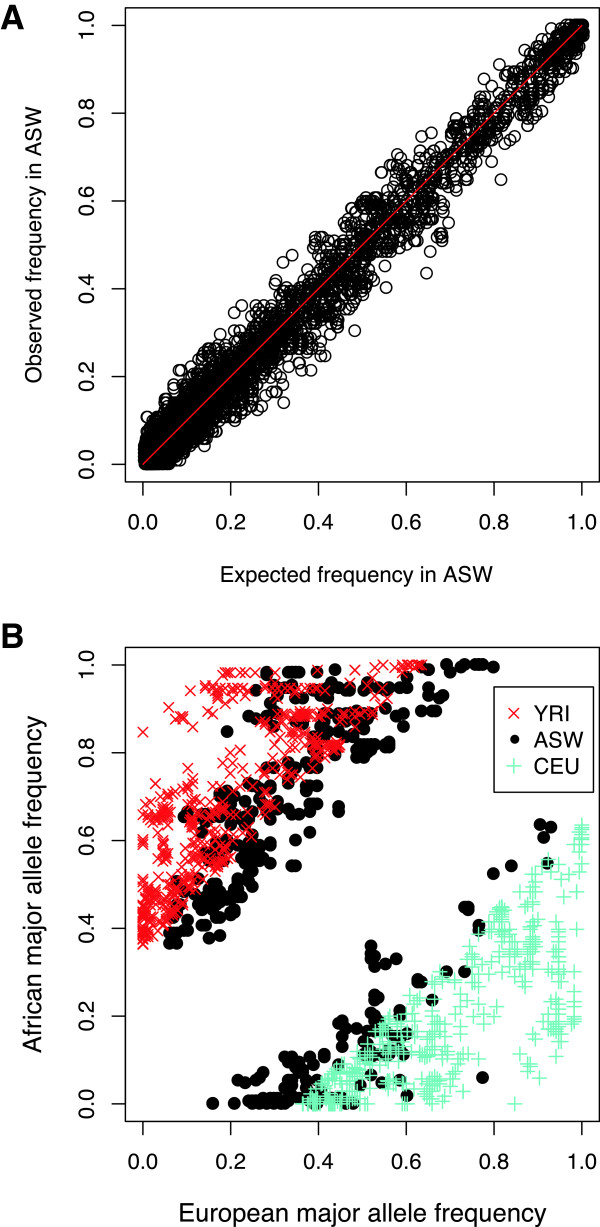
**Allele frequency patterns of 32ADME core genes in African Americans. (A)** A scatter plot of observed vs. expected allele frequencies of 32 ADME core genes in African Americans. **(B)** The allele frequency distribution of 806 highly differential SNPs (a frequency difference larger than 0.37 between at least two populations) among the three populations.

Where *f*^*CEU*^ and *f*^*YRI*^ denote the derived allele frequency of each locus in CEU and YRI, respectively, and *ρ*^*Eur*^ represents the contribution of European ancestry in each locus. As shown in Figure 2A, the observed and expected values calculated from ASW showed high linear correlation, with a Pearson’s correlation coefficient of r > 0.99, and significance of *P <* 10^-15^. The ancestral source populations therefore determined the allele frequencies of ASW in the ADME core genes. The expected heterozygosity of each locus can be directly calculated from the allele frequency [18], and the haplotype diversity is associated with allele frequency and the linkage disequilibrium of each locus [19]. It may therefore be inferred that ancestral source populations also somewhat determine the genetic diversity of ASW.

The admixture resulted in distinct genetic diversity patterns of the African American population compared with its ancestral source populations, especially in regions that were highly different between populations. For example, we extracted the highly differential loci with frequency difference larger than 0.37 between at least two populations (with an empirical *P* value of less than 0.05 over the whole genome), and presented the frequency distribution of those 806 loci in Figure 2B. The data clearly reveal that the alleles of ASW are largely in moderate frequencies. The heterozygosity and haplotype diversity of these highly differentiated regions should therefore be consistently higher for African Americans.

### Comparison of genetic diversity patterns of ADME genes between African Americans and their ancestral source populations

To compare the overall genetic diversity of ADME genes between African American and its ancestral source populations, we calculated the derived allele frequencies of 284 ADME genes with the exons, introns, and up- and down-stream 10 kb regions. In addition, we separated the above regions into 10 kb bins to avoid bias due to the varying lengths of different genes, and then calculated the expected heterozygosity and haplotype diversity of these bins and examined their distributions.

**Figure 3:**
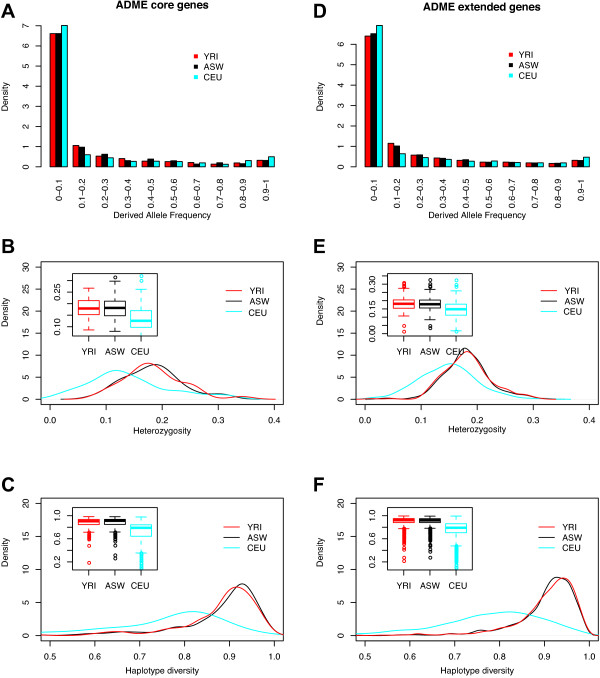
**Genetic diversity patterns of 32 ADME core genes and 252 ADME extended genes. (A)** Derived allele frequency spectra of core genes, **(B)** Expect heterozygosity distributions of core genes, **(C)** Haplotype diversity distributions of core genes, **(D)** Derived allele frequency spectra of extended genes, **(E)** Expect heterozygosity distributions of extended genes, **(F)** Haplotype diversity distributions of extended genes. In these panels, black represents ASW, blue represents CEU, and red represents YRI.

Different genetic diversity patterns of 32 ADME core genes between ASW, CEU, and YRI are shown in Figure 3A-3C. For the 10 frequency bins from 0 to 1, CEU showed an extremely high abundance in the low-derived frequency bin (0–0.1), less abundance at the intermediate frequency bins, but increased abundance in the high-frequency-derived-allele bin (0.9-1; Figure 3A). The unexpected proportions of nearly fixed alleles suggested that natural selection signals were enriched in ADME core genes in the CEU population. Compared with the frequency pattern of CEU, the spectra of the ADME core genes of ASW and YRI showed high levels of similarity, although there are still some differences among many bins, particularly the intermediate frequency bins. For example, ASW showed a higher abundance in the frequency bins of 0.4-0.5, and 0.5-0.6.

Because the alleles with intermediate frequencies were enriched in the ADME core genes from ASW, African Americans exhibited the highest expected heterozygosity compared with the other two populations (Figure 3B). Overall, CEU showed the lowest median heterozygosity value of 0.126, YRI exhibited an intermediate median heterozygosity of 0.179, and ASW demonstrated the highest median heterozygosity (0.181). Hence although the heterozygosity distributions of YRI and ASW were much more similar to each other than to CEU, the curve of heterozygosity in ASW was shifted to higher values than that of YRI (*p <* 0.001), indicating increased genetic diversity due to genetic admixture.

Haplotype diversity analysis of 32 ADME core genes showed similar patterns to the comparison of heterozygosity (Figure 3C). Generally, the haplotype diversity distribution of CEU was lower than the other two populations, the distribution was flatter, and the median value was 0.790. Conversely, the haplotype diversity distributions of ASW and YRI were narrower, and shifted to higher values. When ASW and YRI were compared, ASW had higher haplotype diversity with a median value of 0.912, while the median value of YRI was 0.903 (*p <* 0.001).

When the genetic diversity of 252 extended ADME genes was analyzed (Figure 3D-3F), obvious differences were identified between CEU and the other populations. However, compared with the analysis of the 32 core ADME genes, CEU exhibited a pattern with less enrichment in the very low or high frequency bins (Figure 3D), but shifted to higher values of both heterozygosity (with a median 0.148) and haplotype diversity (with a median 0.792; Figure 3E and 3F). When the 252 extend ADME genes of ASW and YRI were compared, the difference in allele frequency was smaller (Figure 3D). ASW and YRI showed high overlapping heterozygosity and haplotype diversities, and were different only at peak regions of the distributions (Figure 3E and 3F). Specifically, the median heterozygosities were 0.178 and 0.181, while the median haplotype diversities were 0.919 and 0.921, for ASW and YRI, respectively. In the 252 ADME extended genes assessed, ASW therefore showed slightly lower genetic complexity than YRI (*p <* 0.001), in contrast to the results from 32 ADME core genes.

To better characterize genetic architecture of ADME genes, we further compared the genetic diversity patterns of 32 ADME core genes with those of 50 randomly selected genes, and genetic diversity patterns of 252 extended genes with those of 500 randomly selected genes, as well as those of the whole autosomal regions. With respect to derived allele frequency (DAF) spectrums (Additional file 5: Figure S3 A-C), all three populations exhibited an exponential distribution, with CEU showing the highest, ASW moderate, and YRI the lowest enrichment in the low DAF bin (0.0-0.1). With respect to the expected heterozygosity distributions (Additional file 5: Figure S3 D-F), CEU again exhibited the lowest heterozygosity in all the three datasets (two randomly selected and one whole autosomal), while ASW and YRI showed very similar distributions. Similarly, haplotype diversity of CEU was the lowest among the three populations, whereas the distributions of ASW and YRI were comparable, as shown in Additional file 5: Figure S3 G-I. In summary, CEU showed consistently the lowest genetic diversity in all the random data sets we examined, which was consistent with the patterns we observed in ADME genes. However, the genetic diversity of ASW was similar to or even lower than that of YRI in random data sets, which was contrast to the patterns observed in the 32 ADME core genes.

### Characterizing genetic diversity patterns of ADME core genes

**Figure 4:**
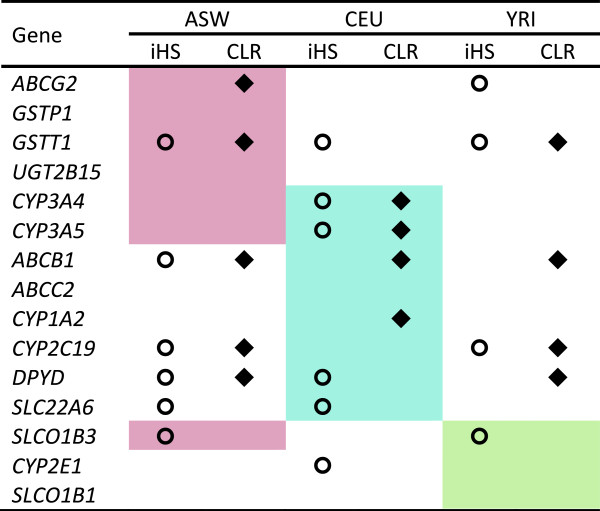
**LSBL analysis and natural selection testing of the ADME core genes.** Brown represents the genes with significant *L*_*ASW*_, blue represents genes with significant *L*_*CEU*_, and green represents significant *L*_*YRI*_. The diamond symbol represents the genes with significant CLR scores, whereas the cycle symbol represents the genes with significant iHS scores.

To investigate why ADME core genes exhibited significantly higher diversity than random datasets, we separately assessed the genetic diversity patterns of population-specific high diversity regions in ASW, CEU, and YRI. The method used to build LSBL (locus specific branch length) trees based on pairwise *F*_*ST*_ values from the three populations were described in Methods. Taking the values of branch length with *P =* 0.01 as a threshold based on the empirical distributions (0.061 for ASW, 0.367 for CEU, and 0.114 for YRI, seen in Additional file 6: Figure S4), we found 15 out of the 32 ADME core genes showing significant LSBL signals. Detailedly, 7 genes (*ABCG2*, *GSTP1*, *GSTT1*, *UGT2B15*, *CYP3A4*, *CYP3A5*, and *SLCO1B3*) showed significant *L*_*ASW*_ fragments, 8 genes (*CYP3A4*, *CYP3A5*, *ABCB1*, *ABCC2*, *CYP1A2*, *CYP2C19*, *DPYD*, *SLC22A6*) displayed significant *L*_*CEU*_ fragments, and 3 genes (*SLCO1B3*, *CYP2E1*, *SLCO1B1*) exhibited significant *L*_*YRI*_ fragments, as shown in Figure 4. In the 10 kb bins spanning entire autosomal regions, as shown in Additional file 7: Figure S5, CEU consistently showed the lowest genetic diversity compared with the other two populations. In contrast, the results of comparison between ASW and YRI depended on the different situations. For example, in significant *L*_*ASW*_ regions, ASW showed lower genetic diversity than YRI, in contrast, whereas in significant *L*_*CEU*_ regions, ASW showed higher single-locus heterozygosity but lower haplotype diversity than YRI, while in significant *L*_*YRI*_ regions, ASW showed consistently higher genetic diversity than YRI. Therefore, the special genetic pattern of ADME core genes is probably due to the prevalence of significant LSBL regions in the core genes, which made the differentiations between ASW and YRI more distinguishable.

Significant LSBL regions within given populations indicate the natural selection signals. Because we were unable to conduct distinct detailed selective sweeps, we used two independent natural selection detection approaches; iHS (integrated Haplotype Score) and CLR (Composite Likelihood Ratio) tests, to validate the selection signals of those genes (Figure 4). In most of genes showing significant LSBL, natural selection signals from iHS and CLR tests were also identified in at least one population, but were not necessarily found in the exact population that exhibited significant LSBL signals. For example, 12 out of 15 genes showed natural selection signals in at least one population by either iHS or CLR. However, only 3 genes (*CYP3A4*, *CYP3A5*, and *CYP1A2*) showed consistent LSBL and iHS/CLR signals in CEU. It is noteworthy that LSBL is a cross-population test, whereas iHS/CLR methods are used for within-population analysis. The inconsistent results observed in Figure 4 therefore accurately explain how natural selection shaped the genetic differences between populations.

Interestingly, 7 of the 15 genes presented in Figure 4 were identified as underlying natural selection by iHS/CLR tests in ASW, which was a similar proportion to CEU (8 out of 15) and YRI (6 out of 15). However, each of the genes with signals in ASW consistently showed similar signals in the ancestral source populations, particularly YRI. For example, *SLC22A6* had underlying selection based on the iHS signal in both ASW and CEU, whereas *ABCG2*, *CYP2C19*, and *SLCO1B3* were identified based on iHS or CLR signals in both ASW and YRI. Finally, *GSTT1*, *ABCB1*, and *DPYD* had underlying selection based on iHS or CLR signals in all populations. The beneficial selective sweeps found in ASW may therefore be inherited from either of its ancestral populations.

Twenty-four of the 252 ADME extended genes exhibited strong LSBL signals in at least one population (Additional file 8: Figure S6). Of these 24 genes, only 13 played a role in natural selection based on iHS/CLR signals, which showed less selective sweeps in the ADME extended genes compared with the core genes. The ADME extended genes also showed much more comparable genetic diversity patterns than the neutral datasets (Figure 3 and Additional file 5: Figure S3), suggesting that genes are subject to less selective pressure compared with the more functionally important ADME core genes.

### Highly differential functional SNPs in ADME genes across the three populations

**Figure 5:**
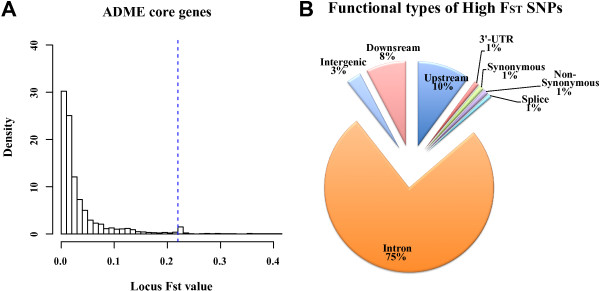
**Analysis of locus-specific differentiation of 32 ADME core genes. (A)** The distribution of the *F*_*ST*_ loci and all sites located in 32 ADME core genes, including regions 10 kb up- and down-stream. In the figure, the dashed line represents the cutoff with an empirical *P* value of 0.01 (*F*_*ST*_ = 0.221). **(B)** The variant effect prediction of highly differentiated loci.

Given the above evidence that some ADME core genes showed natural selection signals in particular populations, it was important to identify the causal mutations that affected the function of the genes. Considering that the potential causal mutations typically exhibit large allele frequency differences between individuals that adapt to the local environment and those that do not, we used global *F*_*ST*_ to identify SNPs with high differential frequencies between populations, and annotated their functions using public datasets. Figure 5A reveals a histogram of *F*_*ST*_ values of all loci from 32 ADME core gene regions (including 10 kb up- and downstream). The ADME core genes showed a significantly higher percentage of mutations with high *F*_*ST*_ (415 out of the total 12,255 SNPs with *F*_*ST*_ >=0.221) compared with 252 ADME extended genes (Additional file 9: Figure S7A), 50 or 500 randomly selected genes (Additional file 9: Figure S7B and C), and the entire autosomal region (Additional file 9: Figure S7D). For the identified highly differential SNPs between the three populations, we used the variance effect predict tools (Ensembl) to predict and catalog the function of each locus (Figure 5B). Out of the SNPs with high *F*_*ST*_ values, 75% were located in introns, 3% were in the intergenic region, 8% in the downstream region, and 10% SNPs were upstream. While the function of the SNPs could not be directly identified, it is possible that they might be associated with regulating gene expression. In addition, three SNPs were found in 3′-UTR regions, three were synonymous mutations, four were non-synonymous mutations, and four were located in splice sites, which combined make up 1% of the total number of SNPs with high *F*_*ST*,_ and are more likely to be directly associated with protein structure and gene expression.

To further explore the function of the SNPs with high *F*_*ST*_, we used two additional functional annotation databases to investigate their detailed function (Table 1). We identified 28 potentially functional SNPs with high *F*_*ST*_ in 11 genes. Of these, *ABCG2*, *CYP1A2*, *CYP3A4*, *CYP3A5*, *SLCO1B3*, and *UGT2B7* had more than one highly differential functional SNPs, showing a natural selection signature (Figure 4). From the 28 highly differential SNPs, 20 were annotated in the PharmGKB database, all of which were associated with drug heterogeneity responses in clinical studies. For example, it was reported that the genotype AA at the SNP rs2032582 in the *ABCB1* gene is associated with an increased required dose of antipsychotics in patients with schizophrenia [20], while CC and AC genotypes are associated with decreased responsiveness to paroxetine in patients with depression compared with the AA genotype [21,22]. Of the remaining eight SNPs, five have been annotated in RegulomeDB, which are significantly associated with gene expression as eQTL. Although the remaining three SNPs have not been annotated in either database, they may still modulate enzyme function as non-synonymous mutations or splice sites in genes. The derived allele frequency and expect heterozygosity of each population for each SNP is listed in Table 1. By comparing the frequencies of the functional SNPs, we found significant differences between ASW, CEU, and YRI. In particular, variants in CEU or YRI were almost fixed to the ancestral (frequency <=0.1) or derived state (frequency >=0.9), while the frequencies of ASW were between those of CEU and YRI. This is consistent with the overall frequency spectrum of the ADME genes, and also explains why the expected heterozygosity in ASW is the highest of the three populations.

**Table 1:** Summary information of highly differential functional SNPs at ADME core genes

**rsID**	**Gene**	**allele**	**f**_**ASW**_	**f**_**CEU**_	**f**_**YRI**_	***He***_**ASW**_	***He***_**CEU**_	***He***_**YRI**_	**Type**	**Dataset**	**Related**
rs2032582	*ABCB1*	C/**A**	0.082	0.453	0.000	0.151	0.496	0.000	NON_SYNONYMOUS	GKB	amitriptyline, atorvastatin, etc.
rs2231164	*ABCG2*	C/**T**	0.287	0.912	0.188	0.409	0.161	0.305	INTRONIC	GKB	antineoplastic agents
rs2622628	*ABCG2*	C/**A**	0.443	0.012	0.494	0.493	0.023	0.500	INTRONIC	GKB	antiepileptics
rs2606345	*CYP1A1*	C/**A**	0.197	0.682	0.006	0.316	0.433	0.011	UPSTREAM	GKB	carbamazepine, phenobarbital, etc.
rs2472304	*CYP1A2*	C/**T**	0.131	0.659	0.000	0.228	0.450	0.000	SYNONYMOUS	GKB	caffeine, paroxetine
rs2470890	*CYP1A2*	G/**A**	0.139	0.659	0.011	0.240	0.450	0.023	INTRONIC	GKB	caffeine, paroxetine
rs2070673	*CYP2E1*	A/**T**	0.369	0.777	0.205	0.466	0.347	0.325	INTRONIC	GKB	cytarabine, ethambutol, etc.
rs12333983	*CYP3A4*	A/**T**	0.361	0.924	0.205	0.461	0.141	0.325	DOWNSTREAM	GKB	tacrolimus
rs3735451	*CYP3A4*	A/**G**	0.279	0.982	0.199	0.402	0.035	0.319	UPSTREAM	GKB	methadone
rs2242480	*CYP3A4*	T/**C**	0.303	0.947	0.142	0.423	0.100	0.244	INTRONIC	GKB	clopidogrel, warfarin, etc.
rs4646437	*CYP3A4*	C/**A**	0.353	0.982	0.256	0.456	0.035	0.381	INTRONIC	GKB	methadone, tacrolimus
rs2687116	*CYP3A4*	C/**T**	0.344	0.982	0.233	0.452	0.035	0.357	UPSTREAM	GKB	tacrolimus
rs2740574	*CYP3A4*	C/**T**	0.344	0.924	0.171	0.452	0.141	0.283	INTRONIC	GKB	carbamazepine, cyclophosphamide, etc.
rs1851426	*CYP3A4*	A/**G**	0.303	0.929	0.171	0.423	0.131	0.283	INTRONIC	GKB	tacrolimus
rs4646458	*CYP3A5*	G/**A**	0.418	0.947	0.278	0.487	0.100	0.402	3PRIME_UTR	GKB	tacrolimus
rs4646457	*CYP3A5*	C/**A**	0.328	0.947	0.159	0.441	0.100	0.268	DOWNSTREAM	GKB	tacrolimus
rs15524	*CYP3A5*	G/**T**	0.648	0.988	0.483	0.456	0.023	0.499	DOWNSTREAM	GKB	tacrolimus
rs7780328	*CYP3A5*	T/**C**	0.336	0.953	0.159	0.446	0.090	0.268	ESSENTIAL_SPLICE_SITE	GKB	alfentanil, alprazolam, etc.
rs776746	*CYP3A5*	G/**A**	0.426	0.959	0.301	0.489	0.079	0.421	INTRONIC	RegulomeDB	-
rs11101985	*GSTM1*	G/**C**	0.098	0.418	0.011	0.177	0.486	0.023	INTRONIC	RegulomeDB	-
rs4630	*GSTT1*	G/**A**	0.279	0.877	0.091	0.402	0.216	0.165	3PRIME_UTR	GKB	dexamethasone, paclitaxel, etc.
rs145334570	*SLCO1B3*	G/**T**	0.516	0.112	0.631	0.499	0.199	0.466	NON_SYNONYMOUS	-	-
rs4762683	*SLCO1B3*	T/**C**	0.516	0.112	0.631	0.499	0.199	0.466	SPLICE_SITE	-	-
rs3764009	*SLCO1B3*	C/**T**	0.484	0.888	0.369	0.499	0.199	0.466	SPLICE_SITE	-	-
rs7311358	*SLCO1B3*	G/**A**	0.484	0.888	0.369	0.499	0.199	0.466	NON_SYNONYMOUS	GKB	docetaxel, mycophenolate mofetil
rs7435827	*UGT2B17*	A/**G**	0.098	0.665	0.046	0.177	0.446	0.087	INTRONIC	RegulomeDB	-
rs7434408	*UGT2B17*	A/**G**	0.172	0.706	0.068	0.285	0.415	0.127	INTRONIC	RegulomeDB	-
rs74764812	*UGT2B17*	T/**C**	0.172	0.665	0.057	0.285	0.446	0.107	INTRONIC	RegulomeDB	-

It is noted that in the column “allele”, the first allele is ancestral state, while the second one highlighted in bold is derived state.

### Functional haplotype analysis of ADME genes between the three populations

In pharmacogenetic studies, the clinical phenotypes of drug metabolism are more likely to be dominated by the haplotype composed of functional variants, rather than single independent SNPs. Based on the PharmGKB database, we therefore analyzed the diversity and distributions of the clinical haplotypes of the ADME core genes between the three populations. Since the nomenclature committee defines the composition of the clinical haplotypes, some variants may only exist in certain individuals and not in healthy samples in the 1000 Genomes Project. As a result, only a partial component of the completed clinical haplotype could be found for 28 of the 32 ADME core genes. Detailed information and the significance of pairwise comparison based on bootstrap resampling are shown in Table 2. In these genes, ASW showed the highest diversity, whereas CEU showed the lowest. For example, ASW had significantly higher haplotype diversity than CEU at 22 genes (*P <* 0.05). In contrast, ASW exhibited comparable haplotype diversity to YRI, and so only six genes were significantly different between ASW and YRI. In five out of the six genes (*ABCG2*, *CYP1A2*, *CYP3A4*, *GSTT1*, and *UGT1A1*), ASW showed higher haplotype diversity, while in *NAT2*, YRI had higher haplotype diversity than ASW (Table 2).

**Table 2:** Haplotype diversity analysis of the 32 ADME core genes

**Gene**	***Hd***_**ASW**_	***Hd***_**CEU**_	***Hd***_**YRI**_	***P* value of 10000 times resampling**		
				**ASW_CEU**	**ASW_YRI**	**CEU_YRI**
*ABCB1*	0.6853	0.7284	0.6655	0.3221	0.6599	0.1000
*ABCC2*	0.6151	0.6966	0.6652	0.0238	0.2121	0.2892
*ABCG2*	0.9678	0.8872	0.9414	0.0000	0.0400	0.0001
*CYP1A1*	0.4050	0.2184	0.3655	0.0200	0.4900	0.0062
*CYP1A2*	0.7904	0.4904	0.6999	0.0000	0.0022	0.0000
*CYP2A6*	0.3781	0.1962	0.4553	0.0200	0.2600	0.0000
*CYP2B6*	0.8594	0.7722	0.8208	0.0021	0.2014	0.1000
*CYP2C19*	0.8328	0.6718	0.8701	0.0000	0.1229	0.0000
*CYP2C8*	0.3333	0.2178	0.3205	0.0800	0.9400	0.0800
*CYP2C9*	0.2800	0.1009	0.3358	0.0028	0.5000	0.0000
*CYP2D6*	0.7261	0.7436	0.7233	0.5936	0.9600	0.4799
*CYP2E1*	0.7009	0.4614	0.7062	0.0000	0.8974	0.0000
*CYP3A4*	0.4552	0.0578	0.3594	0.0000	0.0285	0.0000
*CYP3A5*	0.5461	0.1125	0.4932	0.0000	0.2577	0.0000
*DPYD*	0.4975	0.2847	0.4901	0.0000	0.7000	0.0000
*GSTP1*	0.5182	0.5415	0.4742	0.4746	0.0912	0.0600
*GSTT1*	0.4054	0.2178	0.1662	0.0004	0.0000	0.3400
*NAT1*	0.5198	0.4005	0.5109	0.0035	0.8000	0.0060
*NAT2*	0.8245	0.6897	0.8673	0.0000	0.0283	0.0000
*SLC22A1*	0.6034	0.7277	0.5445	0.0040	0.2492	0.0000
*SLC22A2*	0.2536	0.1619	0.2691	0.1800	0.8000	0.0392
*SLC22A6*	0.1236	0.0000	0.1278	0.0015	0.9338	0.0001
*SLCO1B1*	0.7177	0.6446	0.6918	0.1400	0.5753	0.3198
*SLCO1B3*	0.8954	0.6618	0.9010	0.0000	0.6520	0.0000
*SULT1A1*	0.1378	0.0685	0.1847	0.1505	0.5000	0.0096
*TPMT*	0.6124	0.5236	0.5688	0.0375	0.6000	0.2234
*UGT1A1*	0.7508	0.5671	0.6923	0.0000	0.0049	0.0001
*UGT2B7*	0.7506	0.6741	0.7337	0.0470	0.8000	0.1000

The haplotype diversity values of 32 ADME core genes in three populations, and the significances of haplotype diversity between each two populations were calculated by 10000 times resampling. More detailed haplotype information of each gene can be found in Additional file 10: Table S3.

**Figure 6:**
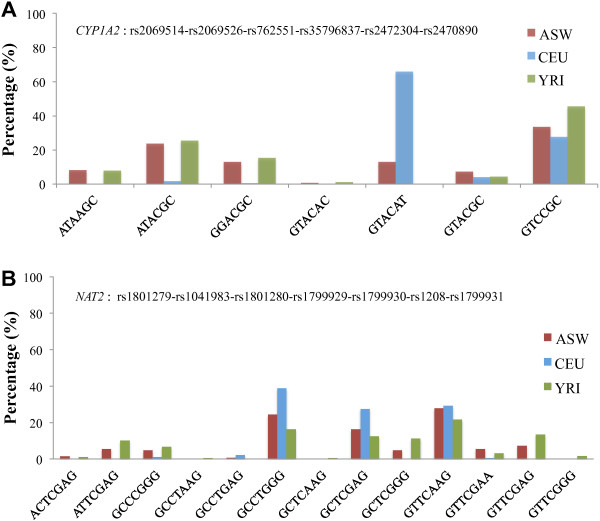
**The haplotype distribution analysis.** The haplotype distribution in three populations: **(A)***CYP1A2* and **(B)***NAT2*.

Two examples of haplotype abundance distributions for *CYP1A2* and *NAT2* are shown (Figure 6). We observed that seven different *CYP1A2* haplotypes (rs2069514, rs2069526, rs762551, rs35796837, rs2472304, and rs2470890) were composed of six SNPs (Figure 6A). Strikingly, the haplotype particularly enriched in CEU is GTACAT (66.9%), which is not observed in YRI, and has a frequency of 13.1% in ASW that may be due to admixture. The haplotype GTACAT is close to the clinically defined haplotypes CYP1A2^*^1 M and ^*^1Q [23]. It can be distinguished from other haplotypes because it carries derived mutations at rs762551 (-163C > A^*^), rs2472304 (2159G > A^*^), and rs2470890 (5347C > T^*^), of which rs2472304 and rs2470890 are associated with rapid metabolism of caffeine and anti-depression drugs such as paroxetine [24,25]. Interestingly, these two SNPs are also in the list of highly differential mutations (Table 1), suggesting that they could be potential causal mutations in the *CYP1A2* gene for local adaptation of Europeans, leading to specific genotypes and haplotypes in CEU. This also explains why ASW showed higher genetic diversity at the *CYP1A2* gene than YRI, since the genetic diversity of the admixed population is shaped by both the influence of the admixture and natural selection. In this example, African Americans inherited some beneficial mutations from one ancestral population (CEU) that do not exist in the other (YRI).

In the haplotype analysis of *NAT2* (Figure 6B), although CEU had the lowest diversity, the haplotypes were distributed into three groups with similar proportions: GCCTGGG (38.8%), GCTCGAG (27.7%), and GTTCAAG (29.4%), which are also common haplotypes in ASW and YRI. For the 13 haplotypes formed by 7 SNPs (Additional file 10: Table S3), 12 haplotypes were found in YRI, while only 10 were identified in ASW. With the exception of the three common haplotypes mentioned above, all other haplotypes exist at low frequency (<10%) in ASW. It is therefore clear that the haplotype diversity of the *NAT2* gene in ASW is lower than in YRI. Considering that we did not find any natural signals of the *NAT2* gene in the three populations here, it is likely that the genetic diversity of *NAT2* in African Americans was mainly influenced by admixture. It is therefore noteworthy that we could not apply the efficacy and safety standard of *NAT2* substrates in African Americans directly to Africans, since Africans show higher genetic diversity in this region.

## Discussion

In this study, we investigated the genetic diversity of drug metabolism-related (ADME) genes in African-Americans (ASW) compared with Europeans (CEU) and Africans (YRI), which are the representative ancestral source populations of African-Americans according to a previous study [17]. As expected, the genetic diversity of the admixed population, such as allele frequency, expected heterozygosity, and haplotype diversity, was largely determined by its ancestral source populations, demonstrating the large influence of admixture on the genetic profiles of African Americans, including drug related genes. In practice, due to few pharmacogenomics studies carried out on African populations, the results from African Americans, which have been more extensively studied, are expected to benefit Africans. However, it is noteworthy that there could be considerable differences of drug responses between African and African American populations. In addition, it was reported that the contribution of African ancestry to African Americans was mainly from west and west-central Africans (~73%) but also from other African populations (~7%) [14]. Therefore, despite taking YRI as representation of Africans sources would not significantly bias the local ancestry inference [17,26], the differences between African American and African populations could be more complicated than what we presented here. Therefore, we suggest it is necessary to make efforts conducting pharmacogenomics studies in African populations in the future.

To further investigate the influence of admixture on the genetic architecture and diversity patterns of African Americans, we performed general genetic diversity comparisons, and found that ASW had a higher genetic complexity than CEU or YRI in the functionally important ADME core genes. It is expected that the ADME genes in ASW populations would have higher genetic diversity than CEU because ancient Europeans were subjected to severe migrational blocks compared with Africans, based on the “out of Africa” theory [27], and thus exhibit lower diversity [28]. Consequently, African Americans received more gene flow from Africans than from Europeans [26,29]. Nevertheless, it was surprising that ASW showed much higher genetic diversity in ADME core genes than YRI, which is significantly different from the patterns observed in the randomly selected genes or whole autosomal regions.

From a comparison of the genetic diversity of ADME core genes across the three populations, ASW showed the highest complexity by the main influence of admixture and enriched selection signatures as complementary. Since these results are based on comparisons of general patterns, these conclusions may not be applied directly to certain cases. We therefore further investigated the genetic diversity of each ADME gene, with particular focus particularly on the highly differentiated SNPs. As with gene-based analysis, CEU showed the lowest genetic complexity in most examples, while ASW showed enriched mediate allele frequencies, higher heterozygosity, and more complex haplotype diversity compared with CEU or YRI in certain genes such as *ABCG2*, *CYP1A2*, and *CYP3A4*. However in some genes such as *NAT2*, ASW showed a lower genetic diversity than YRI.

Due to population admixture, the ASW showed the different allele frequencies from its ancestral source populations, especially, ASW has higher heterozygosity and haplotype diversity than CEU and YRI in some important functional variants or haplotypes of ADME genes (Tables 1 and 2). Differential allele frequencies of the functional variants among populations suggested the phenotypes of drug responses with which these variants are associated could be also different among those populations. Generally speaking, the higher heterozygosity and haplotype diversity indicate that the distribution of phenotypic drug responses is broader in that certain population. For instance, we identified two functional SNPs of *CYP1A2* reported by clinical studies that showed significant differentiation of allele frequencies and heterozygous states among the three populations, while ASW exhibited significantly higher haplotype diversity in *CYP1A2* gene than the other two populations. To our knowledge, so far there has been no systemic study investigating the phenotypic distributions of CYP1A2’s substrates in these three populations, but we thought our observations should benefit exploring the population differentiations of clinic consequences at the genetic level. However, it is noteworthy that the genetic variants are only one of the factors affecting drug responses and most of explicit consequences of genetic variants are not yet fully understood. Thus, the phenotypic consequences of population differentiations of ADME genes should be carefully validated in future studies. On the other hand, although the role of ethnicity in pharmacogenomics studies is still debatable, there are essential ethnic consequences of the different drug dose requirements among different populations [30]. Given that African Americans exhibited higher genetic diversity due to admixture, individual genotyping/sequencing is necessary in the future pharmacogenomic studies of African Americans because higher heterogeneity of drug responses is also expected in admixed populations and any oversimplified ethnic medicine standards might be inappropriate.

In this study, we established the connection between genetic diversity and the effects of clinic drug efficacy and safety based on literatures and public database. Especially, the PharmGKB database provides an opportunity to study the functional consequence of highly differentiated SNPs between different populations using the clinical results manually collected from literature. On the other side, the significant advancement of next generation sequencing and the establishment of public databases such as the 1000 Genomes Project have allowed us to access to the full spectrum of ADME gene mutations among different populations. However, some mutations in PharmGKB are not present in the 1000 Genomes dataset, which may be due to either rare mutations that only exist in some certain patients, or the sequencing depth of the 1000 Genomes Project is not sufficient to detect them.

The genetic diversity patterns between ASW, CEU, and YRI identified in this study could not completely explain the heterogenic drug responses between different populations, but still have important clinical implications. In addition, high-throughput DNA sequencing technology provides additional information not available from traditional pharmacogenetic studies. For example, we discovered eight highly differential SNPs which were not identified in PharmGKB: one non-synonymous SNP, two splice sites, and five intronic SNPs (Table 1). These data may have important functional implications for pharmacogenomics studies.

## Conclusion

Inter-ethnic genetic differences are shaped by both demographic history that affects genome-wide pattern, such as population subdivision and admixture, and evolutionary forces such as natural selection that affect local regions only. In this study, we identified considerable differences between African American and African populations in some functionally important ADME genes, indicating individuals from the two populations should be treated differently in pharmacogenomics. It is likely the genetic characteristics of ADME core genes in African Americans have been shaped by both genetic admixture and natural selection.

## Methods

### Genetic variation data

The investigations of genetic diversity in this study were based on 1000 Genomes project Phase I data [31]. Given the low coverage of sequencing data (2-4x) and even lower coverage on sex chromosomes (1.74x), we focused on the autosomal SNP data in which most of ADME genes are located. We extracted the genetic variation data of African Americans (ASW), Europeans (CEU), and Africans (YRI) from the VCF files released by the 1000 Genomes Project, and the genetic variation data have been already phased with BEAGLEs [32]. The sequencing error in the condition of low coverage could make some singletons unreliable [33] and our work focused on high frequency SNPs, therefore we filtered out the monomorphic sites and singletons in the 234-pooled individuals. Finally, we obtained a total of 18,389,222 SNPs from 61 ASWs, 85 CEUs, and 88 YRIs. Derived allele frequencies and positive selection tests (such as iHS and CLR tests) were only performed on SNPs with known ancestral information that were obtained from the 1000 Genomes Project. As a result, there were a total of 16,224,331 SNPs with known ancestral states, which is approximately 88.2% of the total SNPs obtained.

### ADME genes and putative neutral datasets

As described previously [34], the ADME gene lists were obtained from the PharmaADME database (http://www.pharmaadme.org/), including the core and extended sets [35], as shown in Additional file 4: Table S2. After excluding the genes located on sex chromosomes, there are 32 core ADME genes that play the most important roles in drug metabolism, and 252 extended ADME genes that play a role in drug metabolism, but are not the major factors. Gene coordinate information was obtained from the RefSeq database [36], and 10 kb up- and downstream of each gene was included.

To compare the ADME genes between populations, we used two additional groups of genes/regions as control data. Firstly, to check whether the ADME genes exhibit the specific genetic diversity pattern compared with other coding regions, we created data of several sets of genes (including the 10 kb up- and downstream regions) that were randomly sampled from the RefSeq database without replacement (http://www.ncbi.nlm.nih.gov/RefSeq/). Given the different number of ADME core genes (n = 32) and extended genes (n = 252), we accordingly generated two datasets with comparable number of genes, i.e. 50 and 500 randomly selected genes, respectively. Secondly, data sets were also generated from 10 kb sliding windows in the autosomal regions to compare with ADME genes.

### Functional annotations of SNPs and haplotypes

The functional effects of each SNP from each ADME gene were determined based on the variance effect prediction tools from the Ensembl database [37]. The SNPs that affect gene expression were then studied based on the RegulomeDB dataset [38]. In addition, we studied the SNPs and haplotypes with obvious clinical effects, which were collected and annotated from the PharmGKB database [39].

### Inference of local ancestry

The local ancestry information of ASW was obtained from 1000 Genomes Project (ftp://ftp.1000genomes.ebi.ac.uk/vol1/ftp/phase1/analysis_results/ancestry_deconvolution), which was based on the consistent results of four commonly used methods (LAMP-LD [40], HAPMIX [26], RFMIX [41], and MULTIMIX [42]), and was reported to have high accuracy for ASW (98.9-99.5%) [31]. Briefly, these methods used different principles and algorithms to infer the locus specific ancestry of each individual in admixed populations. For instance, the HAPMIX incorporates background LD to calculate the likelihood of how the haplotypes of admixed individuals relate to those in the ancestral populations, and uses Hidden Markov Model to combine these likelihoods with information from neighboring loci, therefore it could infer an individual's local ancestry, their number of copies of each ancestry at each location in the genome.

From the local ancestry information of ASW (61 individuals in total) in the 1000 Genomes Project, there listed each track of diploid ancestry call for each individual, which including the code of diploid ancestry, the chromosome number, start and end position and length of tract in base pairs. Diploid ancestry calls are a consensus of calls that agree in >=3 of above methods, while the codes of diploid ancestry calls are: 0 is “unknown”, 1 is “European:European”, 2 is “European:African”, and 3 is “African:African”.

### Analysis of genetic diversity

Frequency spectra were constructed by calculating the frequency of derived alleles at each polymorphic site of the genes or regions of interest in a given population. The distributions of heterozygosity and haplotype diversity were calculated in sliding windows of 10 kb, without overlapping across entire genes or regions. To avoid uncertainties in estimations, we excluded windows with less than 5 SNPs. Finally, a total of 168,026 windows were analyzed, among which 227 and 1,797 windows were from the ADME core gene and extended gene sets, respectively, while 381 and 4,092 were from 50 and 500 randomly selected genes, respectively.

The expected heterozygosity (*He*) of each window was calculated using the following formula:

$$H_{e}=2\frac{\sum n_{\mathrm{maj}}\sum n_{\min}}{{\left(\sum n_{\mathrm{maj}}+\sum n_{\min}\right)}^{2}}$$

Where *n*_*maj*_ and *n*_*min*_ are the number of the most and least observed alleles at each locus, respectively.

The haplotype diversity (*Hd*) of each window was calculated using the formula:

$$H_{d}=\frac{N}{N-1}\left(1-\sum x_{i}^{2}\right)$$

Where *N* is the total number of haplotypes, and xi is the frequency of each haplotype. For each ADME core gene, the significance of *Hd* between any two populations was assessed using 10,000 times bootstrap re-sampling [43].

The significance of distributions of heterozygosity and haplotype diversity was assessed using the Kolmogorov-Smirnov test [44], which was implemented in an R script (http://www.r-project.org/).

### Identification of highly differential loci between populations and the detection of natural selection signals in ADME genes

The genetic differences between the three populations at each locus was measured by unbiased *F*_*ST*_ based on Weir and Cockerham [45]. The *F*_*ST*_ for the sliding windows of 10 kb is a weighted average F-statistic over the corresponding loci. For the entire autosomal regions, the top 1 percent of *F*_*ST*_ values for the three populations was 0.221, and thus loci with an *F*_*ST*_ value higher than that were considered to be highly differential SNPs. Next, LSBL (Locus Specific Branch Length) analysis [46] was used to describe the specific differentiation of a given population compared with the other two populations at each locus, by apportioning the genetic diversity into the branch length of a triangular tree. For example, each branch length of ASW could be calculated by

$$L_{\mathrm{ASW}}=\frac{F_{\mathrm{ST}}^{\mathrm{AC}}+F_{\mathrm{ST}}^{\mathrm{AY}}-F_{\mathrm{ST}}^{\mathrm{CY}}}{2}$$

Where $F_{\mathrm{ST}}^{\mathrm{AC}}$, $F_{\mathrm{ST}}^{\mathrm{AY}}$, and $F_{\mathrm{ST}}^{\mathrm{CY}}$ are the pairwise *F*_*ST*_ among ASW, CEU, and YRI, separately. Similarly, the mean LSBL values for the sliding windows of 10 kb were weighted over all loci in the window range. The top 1 percent of the empirical distribution of the average LSBL values of 10 kb windows spanning entire autosomal regions was therefore 0.061 for ASW, 0.367 for CEU, and 0.114 for YRI. The average LSBL value of a given window that is larger than the corresponding threshold was defined as a population-specific significant LSBL region.

The unstandardized iHS scores were calculated using the iHS program [47], and the standardized scores were obtained using Voight’s formula [47], in which the mean and standard deviation of the iHS score in different frequency bins were calculated from all the autosomes, and the frequency bin size was set as 0.01.

CLR (composite likelihood ratio) is a statistic to compute the likelihood ratio of selective sweeps by comparing the spatial distribution of allele frequencies in a given window to the frequency spectrum of null distribution, such as all the autosomal regions. In this study, the SweepFinder [48] program was used to carry out all calculations.

For both iHS and CLR tests, we calculated the standardized iHS or CLR scores of each population for the entire autosomal regions, and used the values with an empirical *P* value of 0.01 as the cutoff to detect natural selection signals at given ADME genes by these two approaches independently.

## Competing interests

The authors declare that they have no competing interests.

## Authors’ contributions

SX conceived the study. SX and JL designed the study. JL, XL, CZ, and LT collected data and performed the analyses. SX, JL, and XL interpreted the data and wrote the paper. All authors have read and approved the final version of the manuscript.

## Supplementary Material

Additional file 1: Figure S1 Local ancestral origins of African Americans. In the figure, rectangular boxes represent 22 autosomes. In each box there are 122 rows representing the diploid sequences of 61 individuals, in which blue color fragments mean European origin, red means originating from Africa, and gray means an unknown component. The start and end positions of 32 ADME core genes are plotted at corresponding locations using green bars. Click here for file

Additional file 2: Figure S2 The local ancestral origins of ASW and corresponding heterozygosity and haplotype diversity variants for 32 ADME core genes. Each box including the 100 kb up- and down-stream regions surrounding the genes. In detail, the local ancestral origins of 61 African Americans for 32 ADME core genes are extracted from Figure S1, and the local heterozygosity and haplotype diversity of the three populations at sliding 10 kb windows were plotted at the corresponding positions. Click here for file

Additional file 3: Table S1 The diploid ancestry tract code for 61 African Americans at 32 ADME genes. In this table, 0 is “unknown”, 1 is “European:European”, 2 is “European:African”, and 3 is “African:African”. Click here for file

Additional file 4: Table S2 The local European ancestral inference for (A) 32 ADME core genes and (B) 252 ADME extended genes. Click here for file

Additional file 5: Figure S3 The genetic diversity patterns for 50 or 500 randomly selected genes and whole autosomal regions. (A)-(C) Derived allele frequency spectra, heterozygosity distribution, and haplotype diversity distribution of 50 randomly selected genes. (D)-(F) The diversity patterns of 500 randomly selected genes. (G)-(I) The diversity patterns for whole autosomal regions. Click here for file

Additional file 6: Figure S4 The LSBL analysis. (A) the LSBL tree constructed by the median values of pairwise *F*_*ST*_ values from the distribution of whole autosomal regions, (B) the distribution of *L*_*ASW*_, (C) the distribution of *L*_*CEU*_, (D) the distribution of *L*_*YRI*_. Note that the dashed lines in Figures S4B, S4C and S4D represent the top 1% of empirical distributions. Click here for file

Additional file 7: Figure S5 The genetic diversity patterns for population-specific significant LSBL regions. (A)-(C) Diversity patterns for significant *L*_*ASW*_ regions. (D)-(F) Diversity patterns for significant *L*_*CEU*_ regions. (G)-(I) Diversity patterns for significant *L*_*YRI*_ regions. Click here for file

Additional file 8: Figure S6 The LSBL analysis and natural selection tests for ADME extended genes. Click here for file

Additional file 9: Figure S7 The loci *F*_*ST*_ distributions. (A) 252 ADME extended genes, (B) 50 randomly selected genes, (C) 500 randomly selected genes, and d) whole autosomal regions. The dashed lines on each panel represent the top 1% of empirical distributions of the whole autosomal region (*F*_*ST*_ value is 0.221). Click here for file

Additional file 10: Table S3 The distribution of clinically defined haplotypes for 28 ADME core genes. Click here for file
