# Supplementary figures and images for: Increased genetic diversity of ADME genes in African Americans compared with their putative ancestral source populations and implications for Pharmacogenomics
Source: BMC Genet. 2014 May 1;15:52. doi: 10.1186/1471-2156-15-52 (PMC4021503; doi:10.1186/1471-2156-15-52)

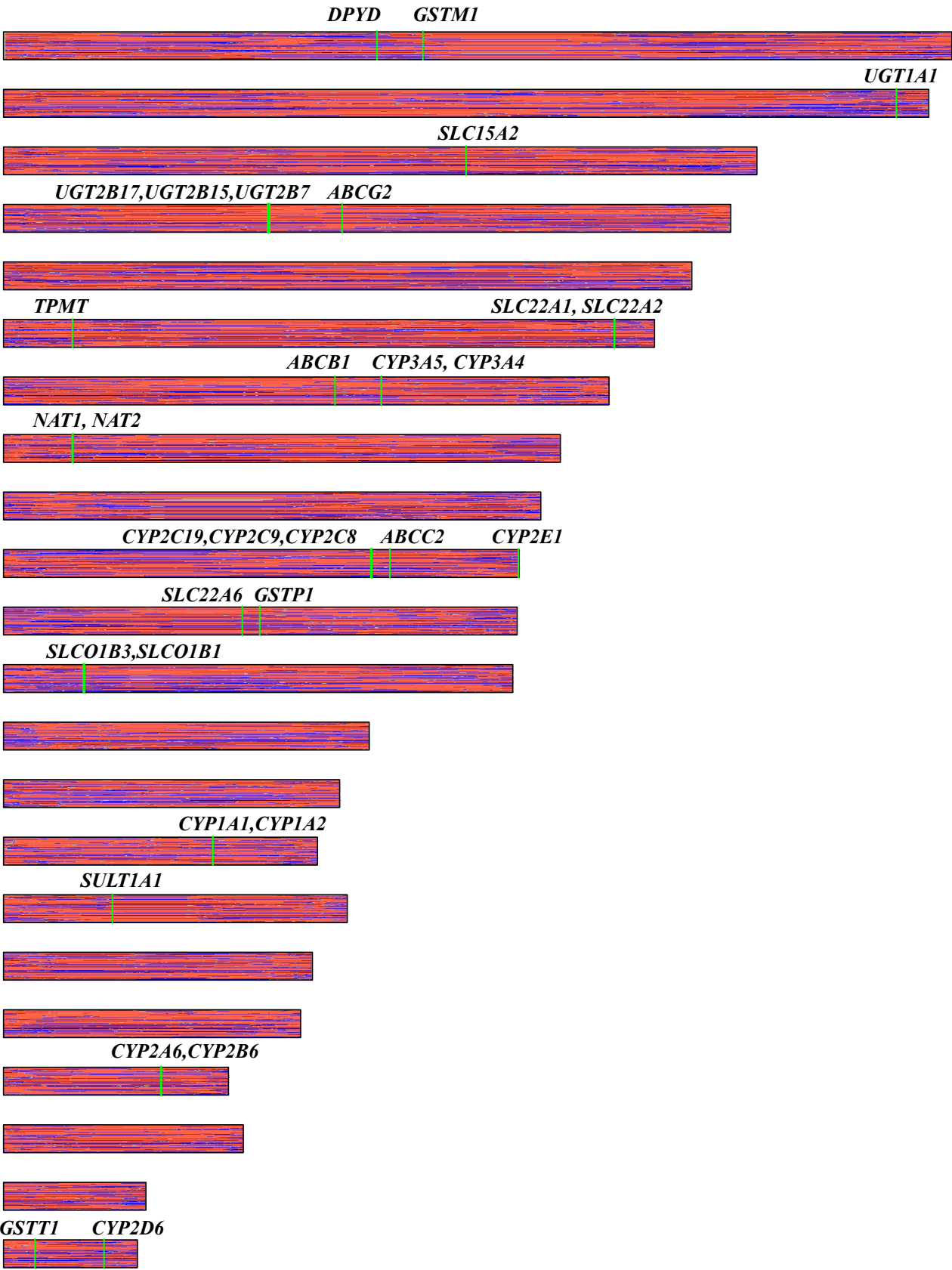

Supplement: Additional file 1: Figure S1 — Local ancestral origins of African Americans. In the figure, rectangular boxes represent 22 autosomes. In each box there are 122 rows representing the diploid sequences of 61 individuals, in which blue color fragments mean European origin, red means originating from Africa, and gray means an unknown component. The start and end positions of 32 ADME core genes are plotted at corresponding locations using green bars. [file 1471-2156-15-52-S1.pdf]

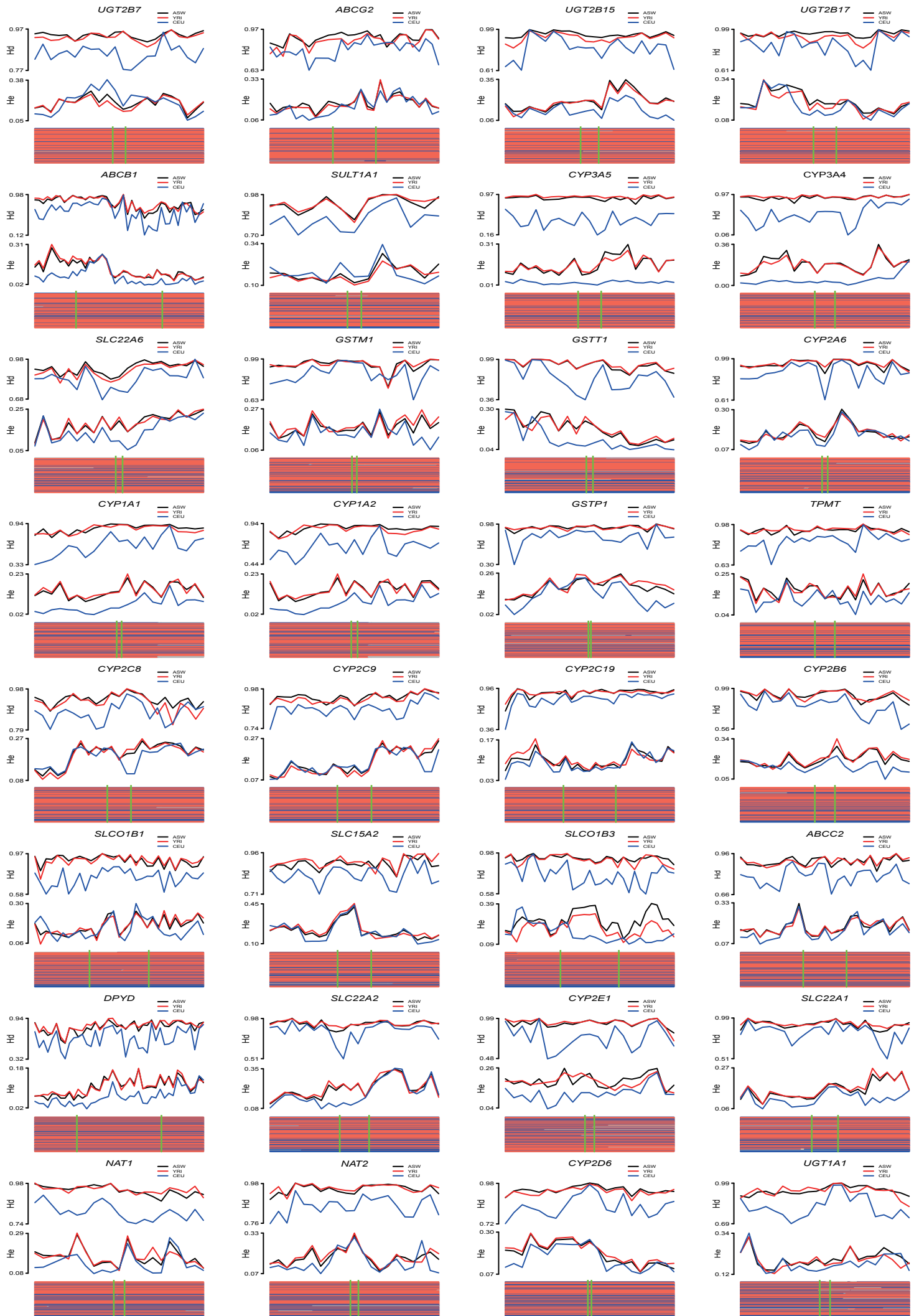

Supplement: Additional file 2: Figure S2 — The local ancestral origins of ASW and corresponding heterozygosity and haplotype diversity variants for 32 ADME core genes. Each box including the 100 kb up- and down-stream regions surrounding the genes. In detail, the local ancestral origins of 61 African Americans for 32 ADME core genes are extracted from Figure S1, and the local heterozygosity and haplotype diversity of the three populations at sliding 10 kb windows were plotted at the corresponding positions. [file 1471-2156-15-52-S2.pdf]

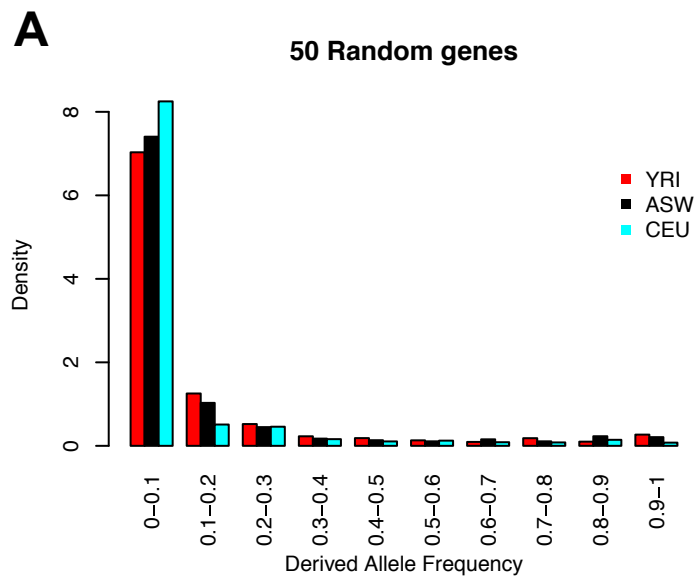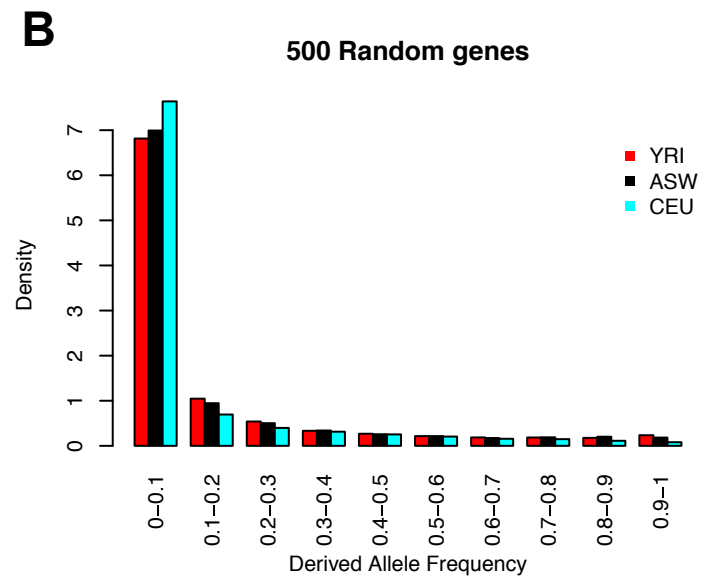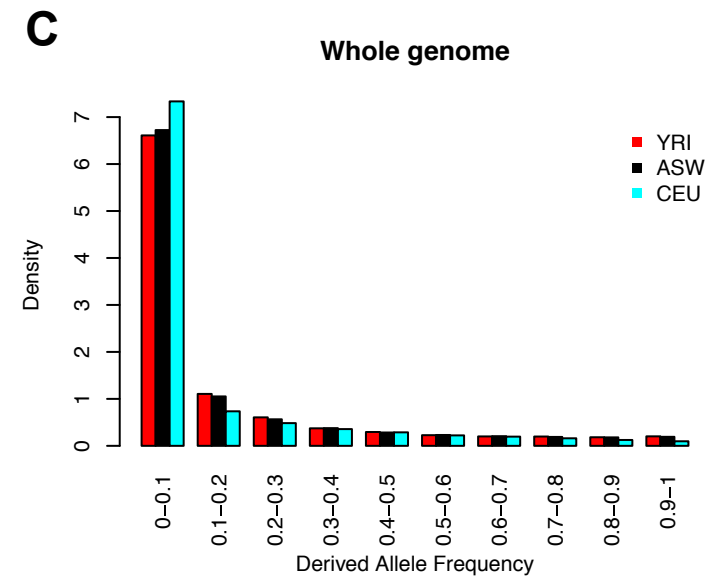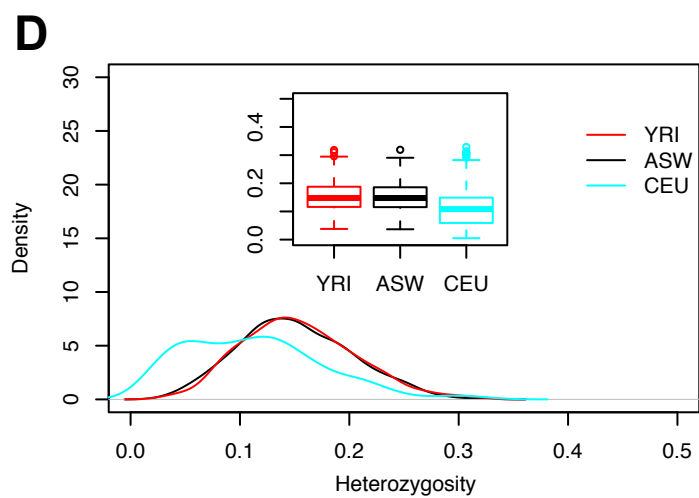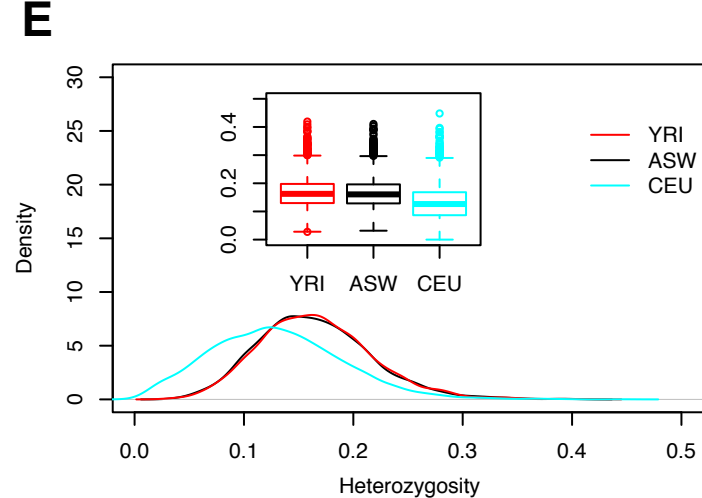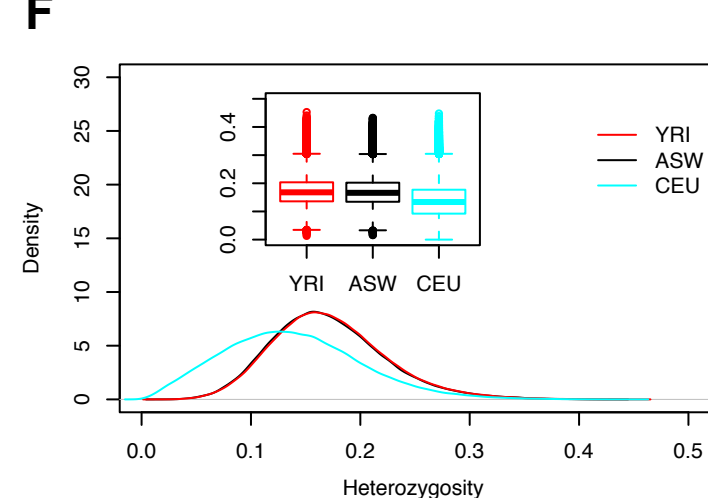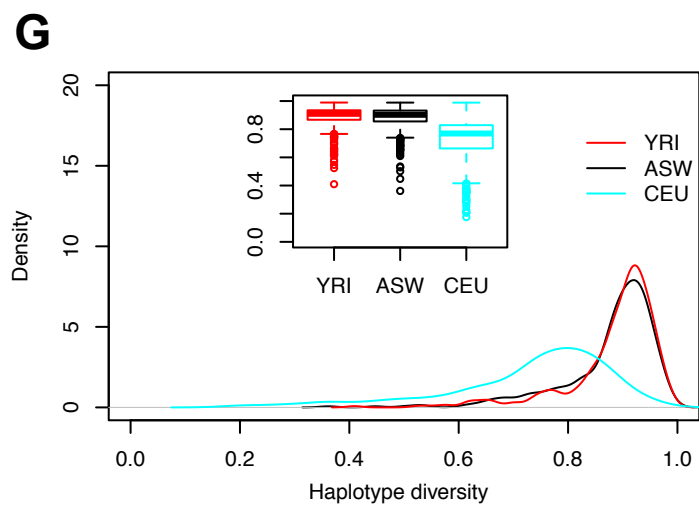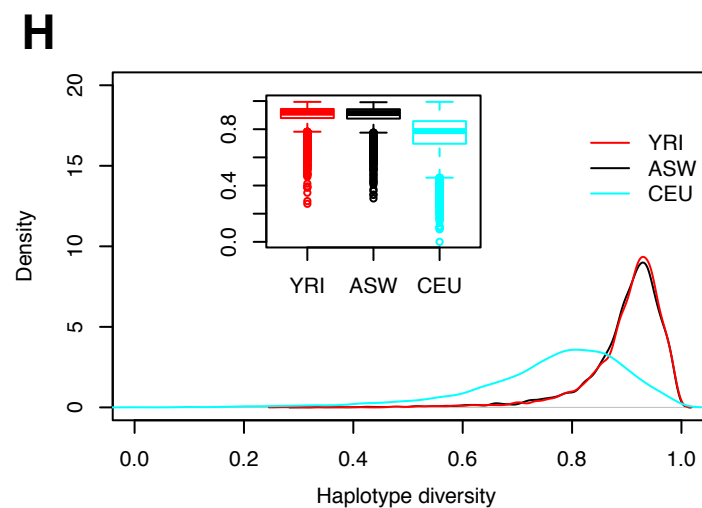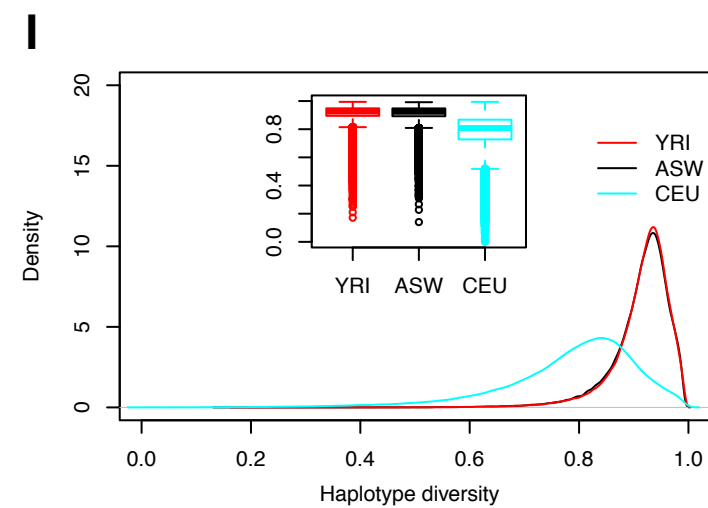

Supplement: Additional file 5: Figure S3 — The genetic diversity patterns for 50 or 500 randomly selected genes and whole autosomal regions. (A)-(C) Derived allele frequency spectra, heterozygosity distribution, and haplotype diversity distribution of 50 randomly selected genes. (D)-(F) The diversity patterns of 500 randomly selected genes. (G)-(I) The diversity patterns for whole autosomal regions. [file 1471-2156-15-52-S5.pdf]

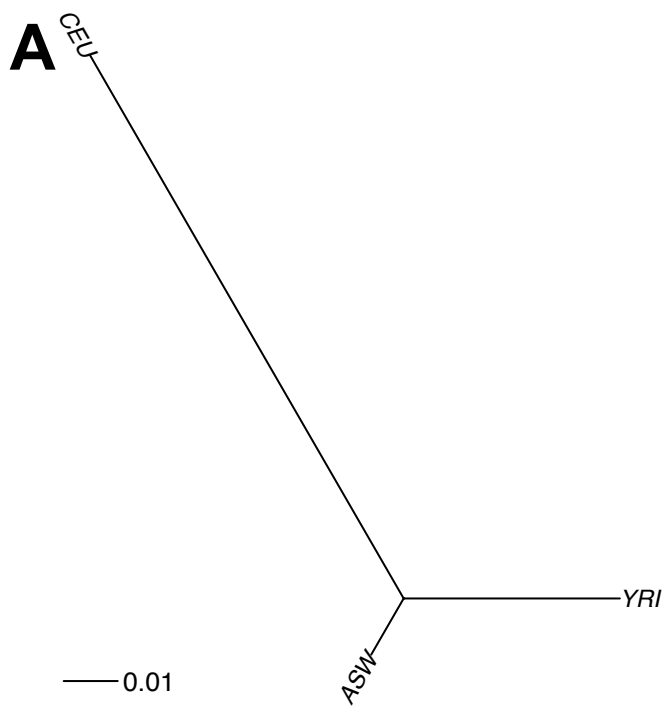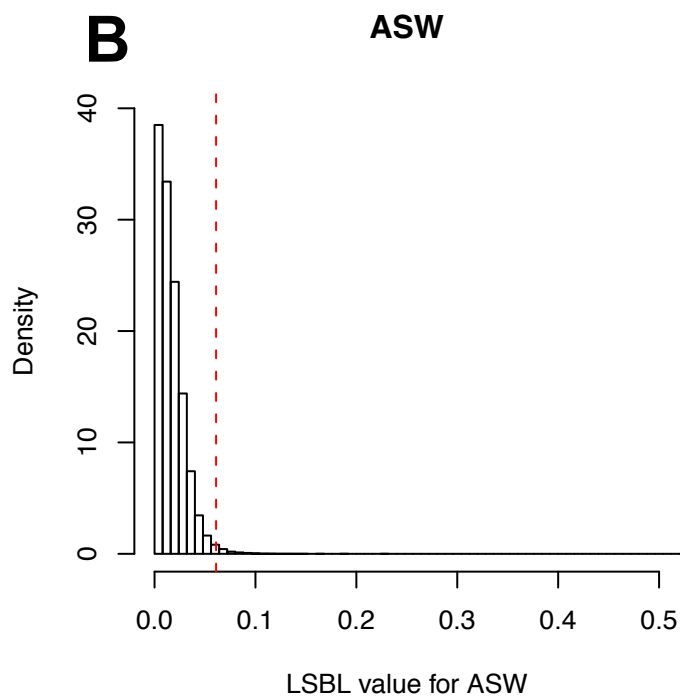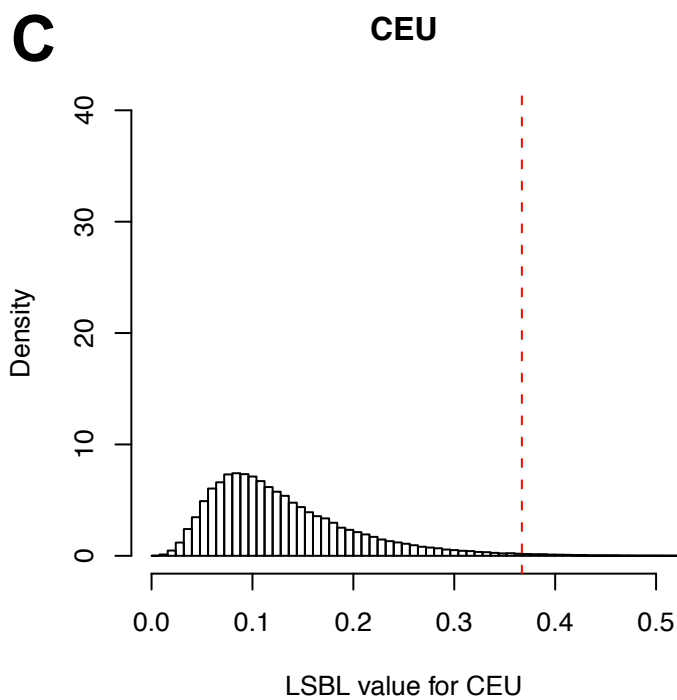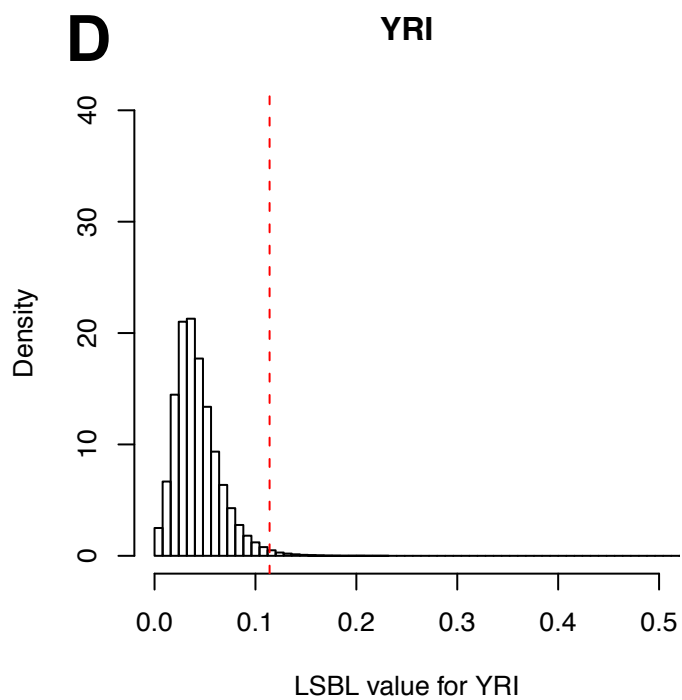

Supplement: Additional file 6: Figure S4 — The LSBL analysis. (A) the LSBL tree constructed by the median values of pairwise FST values from the distribution of whole autosomal regions, (B) the distribution of LASW, (C) the distribution of LCEU, (D) the distribution of LYRI. Note that the dashed lines in Figures S4B, S4C and S4D represent the top 1% of empirical distributions. [file 1471-2156-15-52-S6.pdf]

**A** Significant *LASW* Regions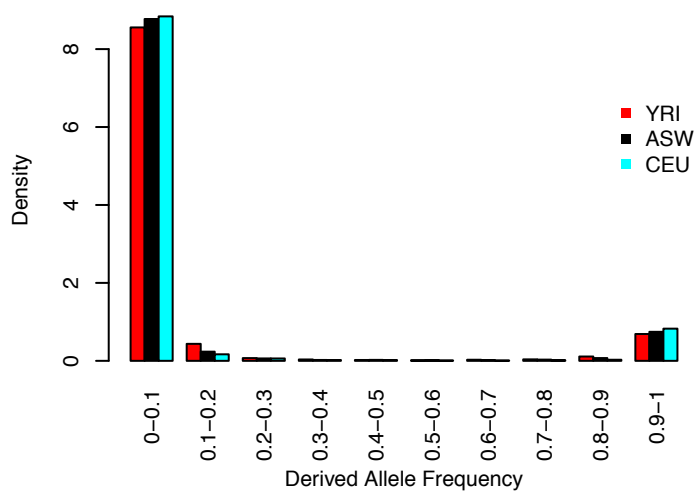**D** Significant *LCEU* Regions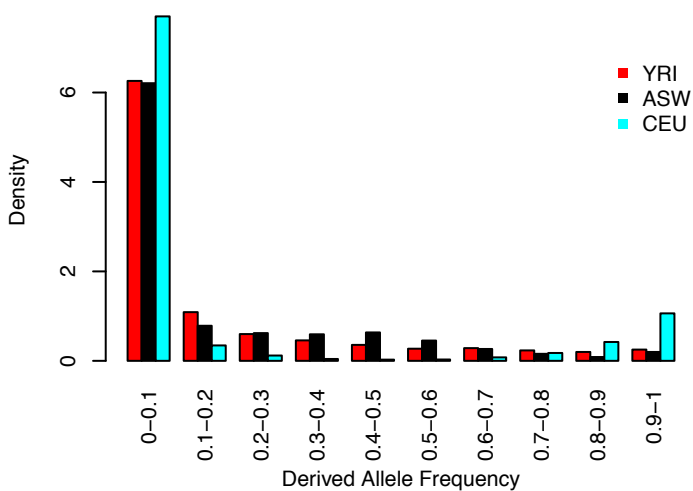**G** Significant *LYRI* Regions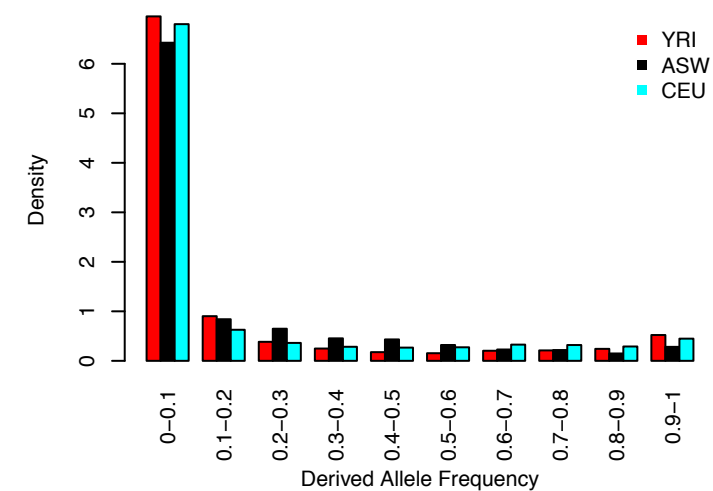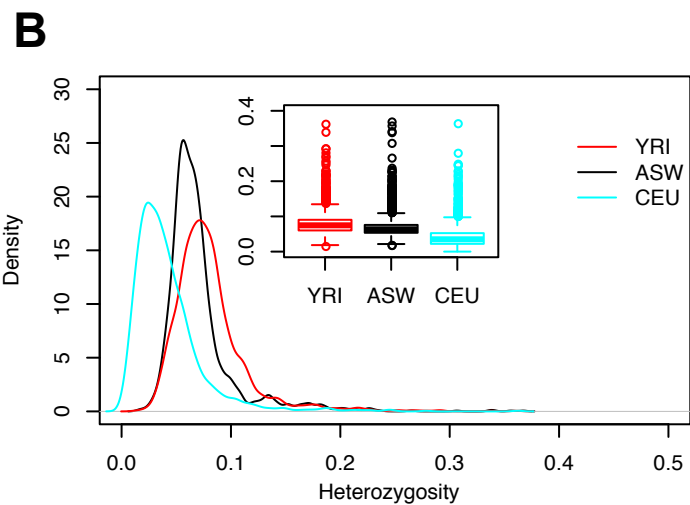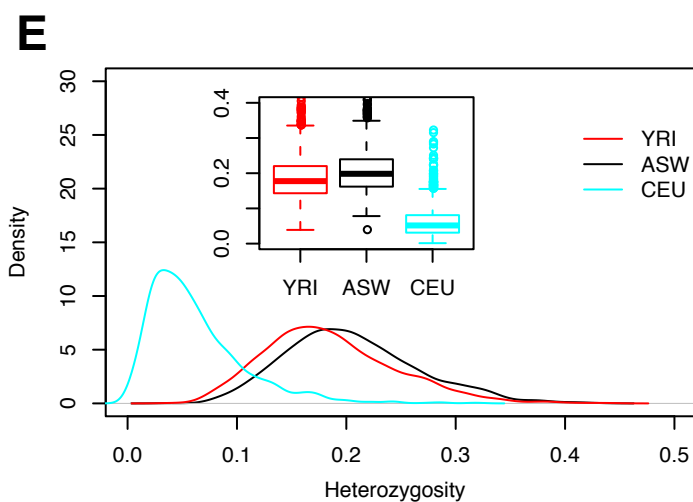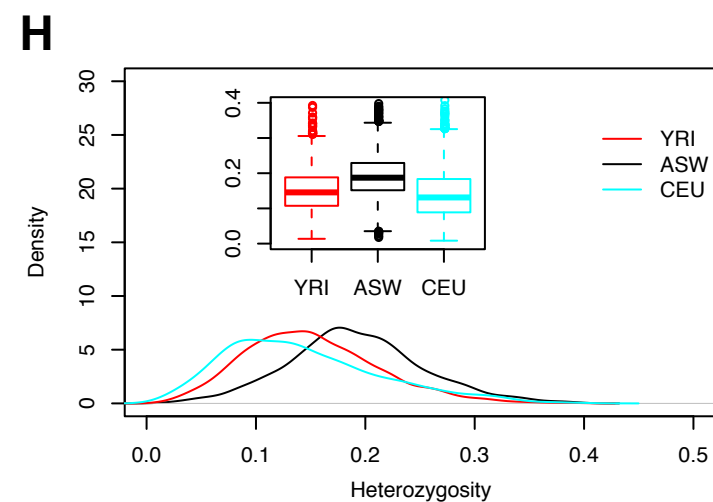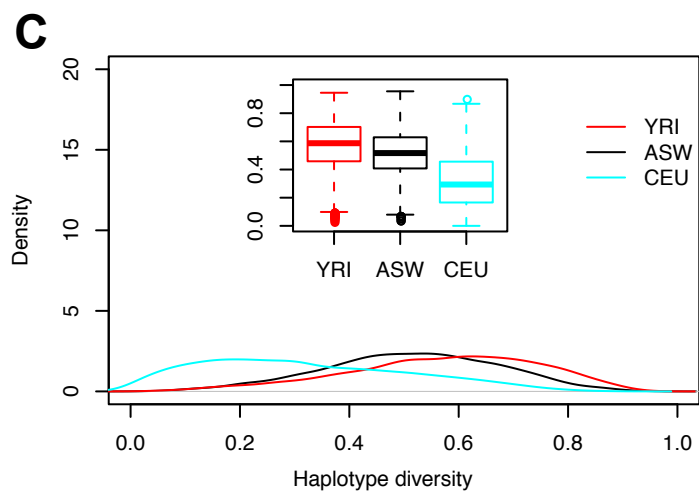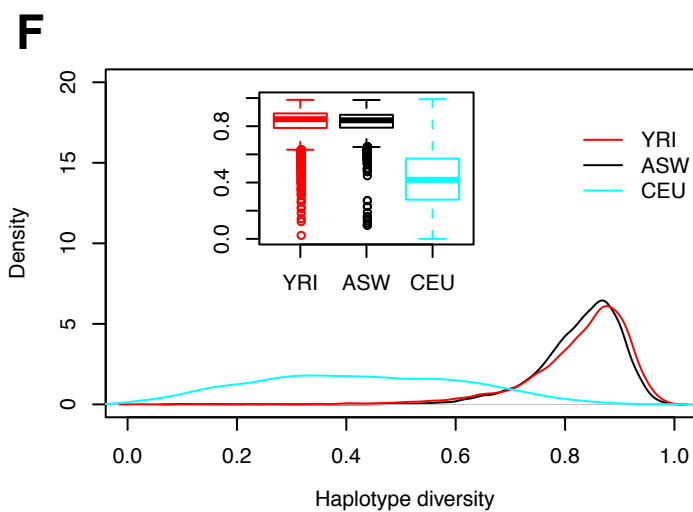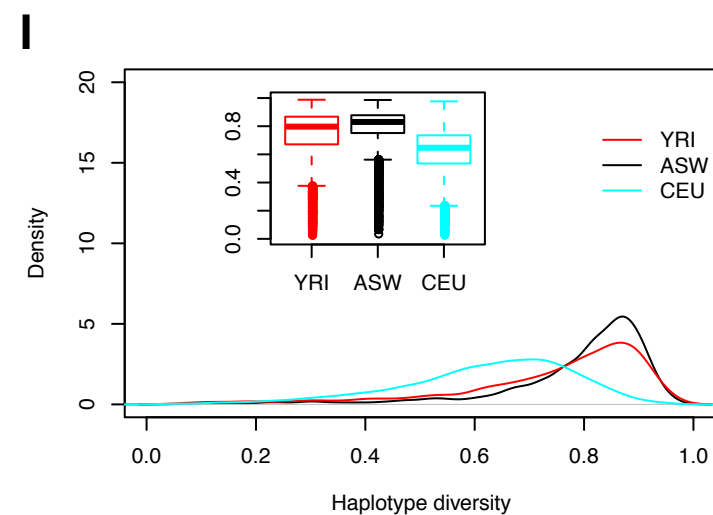

Supplement: Additional file 7: Figure S5 — The genetic diversity patterns for population-specific significant LSBL regions. (A)-(C) Diversity patterns for significant LASW regions. (D)-(F) Diversity patterns for significant LCEU regions. (G)-(I) Diversity patterns for significant LYRI regions. [file 1471-2156-15-52-S7.pdf]

**A****ADME 252 extended ggenes**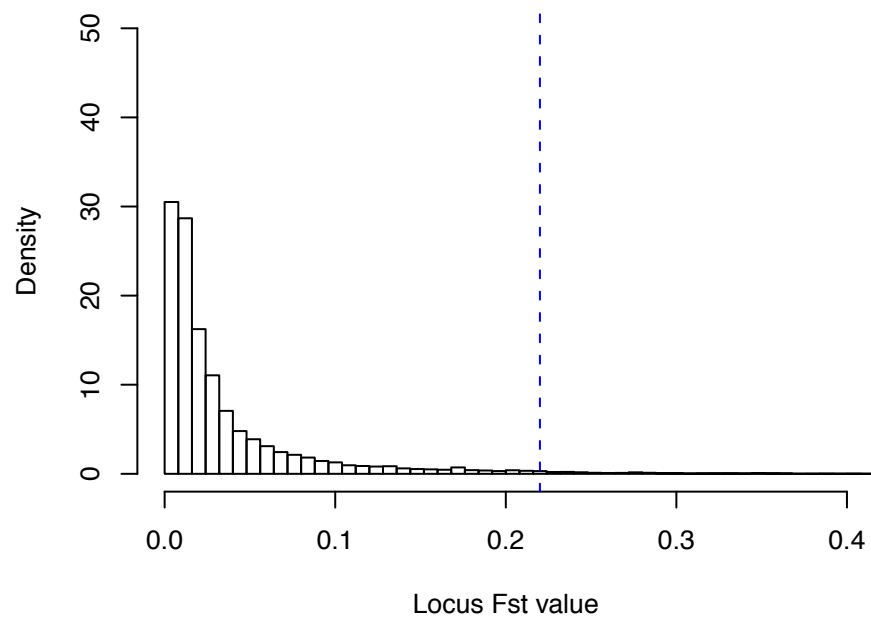**B****Random 50 ggenes**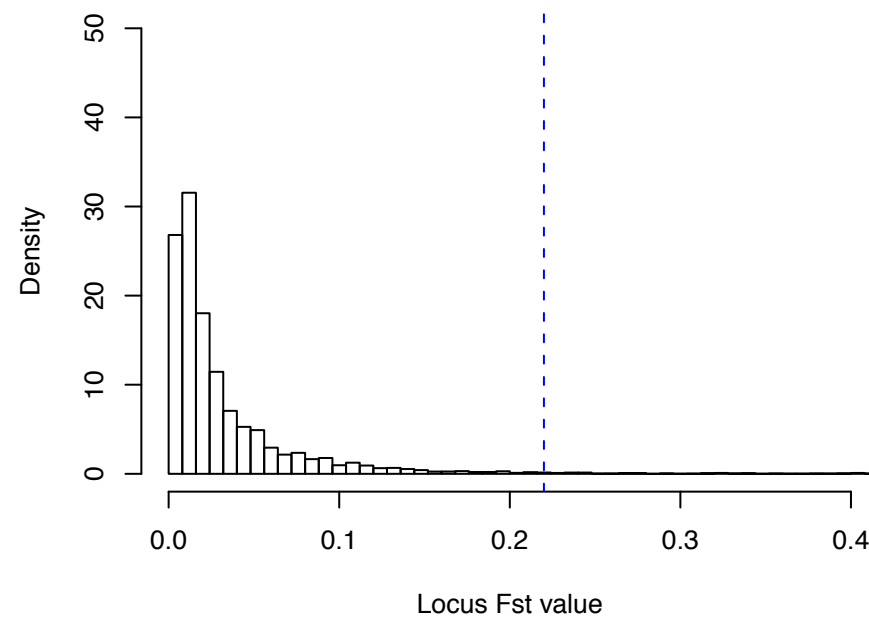**C****Random 500 ggenes**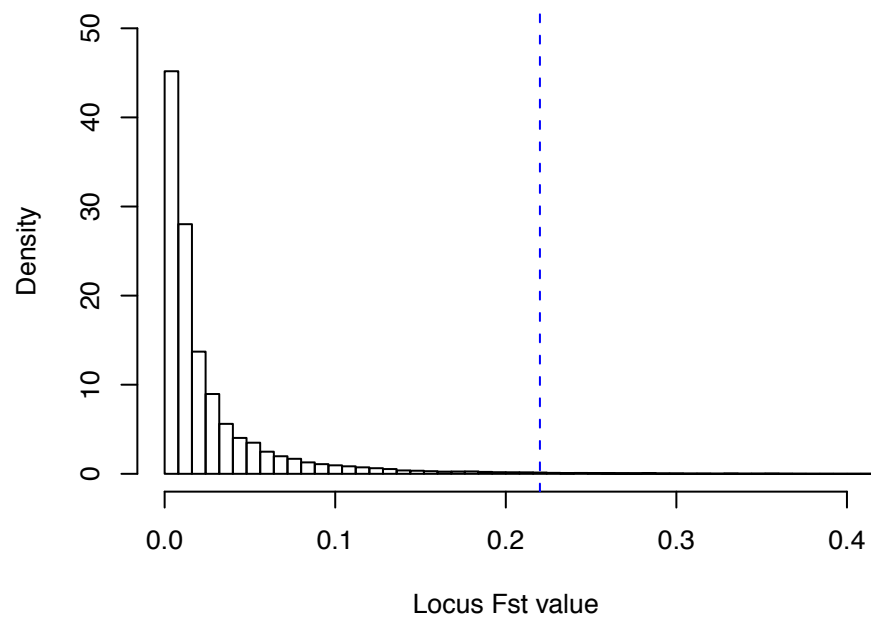**D****Whole autosomal region**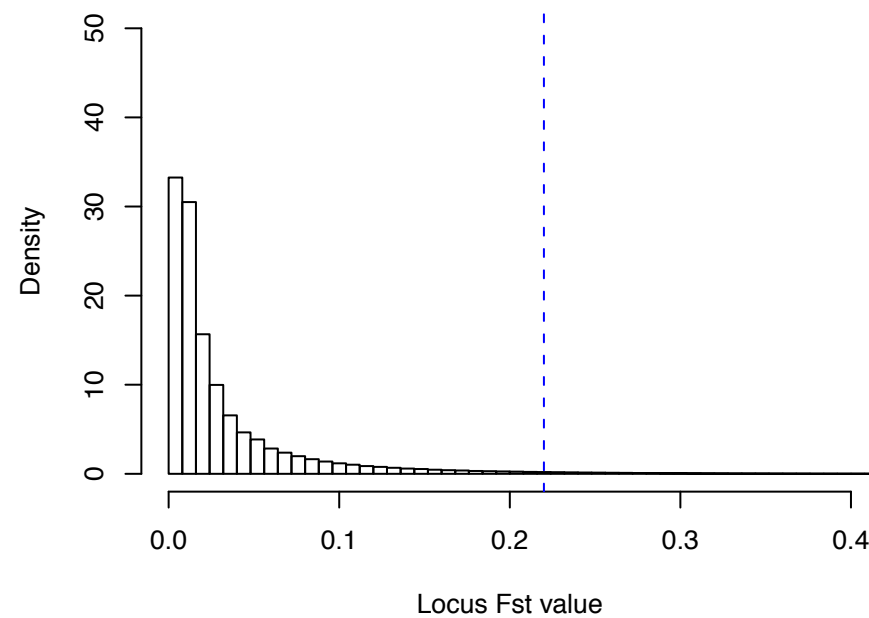

Supplement: Additional file 9: Figure S7 — The loci FST distributions. (A) 252 ADME extended genes, (B) 50 randomly selected genes, (C) 500 randomly selected genes, and d) whole autosomal regions. The dashed lines on each panel represent the top 1% of empirical distributions of the whole autosomal region (FST value is 0.221). [file 1471-2156-15-52-S9.pdf]
