# Supplementary material for: Increased genetic diversity of ADME genes in African Americans compared with their putative ancestral source populations and implications for Pharmacogenomics
Source: BMC Genet. 2014 May 1;15:52. doi: 10.1186/1471-2156-15-52 (PMC4021503; doi:10.1186/1471-2156-15-52)
Supplement: Additional file 8: Figure S6 — The LSBL analysis and natural selection tests for ADME extended genes. [file 1471-2156-15-52-S8.pdf]

Gene	ASW		CEU		YRI	
	iHS	CLR	iHS	CLR	iHS	CLR
DHRS4	○					
CYP3A43						
CYP3A7				◆		
DHRS7					○	
GSR						
MGST2						
PPARD			○	◆		
SLC28A2	○					
GSTK1		◆				◆
ABCC12				◆		
ABCC9	○	◆			○	◆
CYP2C18						
CYP2R1						◆
SLC13A1						◆
SLCO5A1						◆
UGT2B10				◆		
ABCC8					○	
ALDH1A1						
CFTR					○	
CHST5						
DHRS13						
PDE3A						
SLC22A17						
SLCO1C1						
